# Influenza Vaccination Coverage Among Health-Care Personnel — United States, 2012–13 Influenza Season

**Published:** 2013-09-27

**Authors:** Sarah W. Ball, Sara M.A. Donahue, David Izrael, Deborah K. Walker, Charles DiSogra, Rachel Martonik, Stacie M. Greby, Anup Srivastav, Jun Zhang, Peng-jun Lu, Walter W. Williams, Megan C. Lindley, Samuel B. Graitcer, Carolyn B. Bridges, James A. Singleton, Marie A. de Perio, A. Scott Laney

**Affiliations:** Abt Associates Inc., Cambridge, Massachusetts; Abt SRBI, New York, New York; Immunization Svcs Div, National Center for Immunization and Respiratory Diseases; Div of Surveillance, Hazard Evaluations, and Field Studies; Div of Respiratory Disease Studies, National Institute for Occupational Safety and Health, CDC

Routine influenza vaccination of health-care personnel (HCP) every influenza season can reduce influenza-related illness and its potentially serious consequences among HCP and their patients (1–5). To protect HCP and their patients, the Advisory Committee on Immunization Practices (ACIP) recommends that all HCP be vaccinated against influenza during each influenza season (5). To estimate influenza vaccination coverage among HCP during the 2012–13 season, CDC conducted an opt-in Internet panel survey of 1,944 self-selected HCP during April 1–16, 2013. This report summarizes the results of that survey, which found that, overall, 72.0% of HCP reported having had an influenza vaccination for the 2012–13 season, an increase from 66.9% vaccination coverage during the 2011–12 season (6). By occupation type, coverage was 92.3% among physicians, 89.1% among pharmacists, 88.5% among nurse practitioners/physician assistants, and 84.8% among nurses. By occupational setting, vaccination coverage was highest among hospital-based HCP (83.1%) and was lowest among HCP at long-term care facilities (LTCF) (58.9%). Vaccination coverage was higher for HCP in occupational settings offering vaccination on-site at no cost for one (75.7%) or multiple (86.2%) days compared with HCP in occupational settings not offering vaccination on-site at no cost (55.3%). Widespread implementation of comprehensive influenza vaccination strategies that focus on improving access to vaccination services is needed to improve HCP vaccination coverage. Influenza vaccination of HCP in all health-care settings might be increased by providing 1) HCP with information on vaccination benefits and risks for themselves and their patients, 2) vaccinations in the workplace at convenient locations and times, and 3) influenza vaccinations at no cost (7,8).

To provide end-of-season estimates of influenza vaccination coverage among HCP before the 2013–14 influenza season, CDC conducted an opt-in Internet panel survey during April 1–16, 2013.* Two opt-in Internet panel source populations were recruited for the survey through e-mails and website messages. HCP were eligible for the survey if they reported any patient contact. Professional HCP (physicians, nurse practitioners, physician’s assistants, nurses, dentists, pharmacists, allied health professionals, technicians, and technologists) were recruited from the current membership roster of Medscape, a medical website managed by WebMD Health Professional Network. Persons in other HCP occupations (e.g., assistants, aides, administrators, clerical support workers, janitors, food service workers, and housekeepers) were recruited for a health survey from SurveySpot, a general population Internet panel operated by Survey Sampling International that provides its members with online survey opportunities in exchange for nominal incentives.† Among the 2,099 HCP who entered the two panel survey sites and completed the screening questions, 2,005 (95.5%) completed the survey. Of the 1,944 participants whose responses indicated that they worked in a health-care setting or were likely to have contact with patients, 1,469 (75.6%) were professional HCP and 475 (24.4%) were other HCP. §

Survey items included demographic characteristics, occupation type, occupational setting, self-reported influenza vaccination, and employer vaccination policies (vaccination requirements, vaccination available at no cost, and promotion of vaccination [including recognition, rewards, compensation, and free or subsidized vaccination]). Based on responses to the questionnaire, occupation type for HCP from both opt-in Internet panel sources were divided into six groups for this analysis: physicians, nurse practitioners/physician assistants, nurses, pharmacists, other clinical HCP, and nonclinical HCP. Occupational settings for HCP from both opt-in Internet panel sources were divided into four groups for this analysis: hospital, ambulatory/physician office, LTCF, and other clinical setting.¶ Sampling weights were calculated based on each occupation type by age, sex, race/ethnicity, occupational setting, and census region to represent the U.S. population of HCP. Vaccination coverage estimates from opt-in Internet panel surveys completed in 2010–11, 2011–12, and 2012–13 were compared to assess trends over time (6,9). Because the study sample was based on HCP from opt-in Internet panels rather than probability samples, no statistical tests were performed.** Differences were noted when there was a difference of ≥5 percentage points between any values being compared. Data from the 2012–13 influenza season opt-in Internet panel survey were compared with data from comparable opt-in Internet panel surveys conducted during the 2010–11 and 2011–12 influenza seasons.

Overall, 72.0% of HCP reported having had an influenza vaccination for the 2012–13 season, an increase from 63.5% and 66.9% reported in similar opt-in Internet surveys in the 2010–11 and 2011–12 seasons, respectively (Table, Figure 1). Increases were seen within all occupational settings over the three seasons, except for vaccination coverage in LTCF, which was highest (64.4%) during the 2010–11 season, decreased during the 2011–12 (52.0%), and then increased during the 2012–13 season (58.9%) (Table, Figure 2). By occupation type, vaccination coverage was 92.3% among physicians, 89.1% among pharmacists, 88.5% among nurse practitioners/physician assistants, 84.8% among nurses, 68.6% among other clinical personnel, and 64.8% among nonclinical personnel (Table). Vaccination coverage was 83.1% among HCP working in hospitals and 58.9% among those working in LTCFs (Table, Figure 2).

What is already known on this topic?

To help reduce influenza-related morbidity and mortality that occurs in health-care settings, the Advisory Committee on Immunization Practices recommends annual influenza vaccination for all health-care personnel (HCP). Estimates of overall HCP vaccination coverage were 63.5% for the 2010–2011 season and 66.9% for the 2011–12 season.

What is added by this report?

For the 2012–13 season, influenza vaccination coverage among HCP was assessed using an opt-in Internet panel survey of 1,944 self-selected HCP. Overall coverage was 72.0%. Only two HCP groups had vaccination coverage >90%: HCP in facilities with a vaccination requirement had vaccination coverage of 96.5%, and among the individual occupational groups, physicians had vaccination coverage of 92.3%. Vaccination coverage among HCP in long-term care facilities was lower than in other occupational settings. Offering vaccination at no cost on multiple days was associated with higher vaccination coverage.

What are the implications for public health practice?

Comprehensive, work-site intervention strategies that include education, promotion, and easy access to vaccination at no cost can increase HCP vaccination coverage.

Among HCP reporting that their employer required them to receive influenza vaccination, overall vaccination coverage was 96.5%, with coverage above 95% in all occupational settings, including LTCFs (95.8%). Vaccination coverage was 76.9% among HCP who worked in facilities where employers promoted but did not require vaccination (range: 67.0% [LTCFs] to 85.7% [other clinical settings]) and 50.4% among HCP who worked in facilities where employers neither had a vaccination requirement nor promoted vaccination (range: 45.0% [LTCFs] to 67.7% [hospitals]) (Table). Overall, 71.1% of vaccinated HCP reported receiving the vaccination in the workplace. Vaccination coverage among HCP working in facilities that made vaccination available at no cost for >1 day was 86.2% (range: 79.4% [LTCFs] to 88.8% [ambulatory care or physician offices]) compared with 75.7% in facilities that made vaccination available at no cost for 1 day (range: 63.0% [LTCFs] to 87.6% [other clinical settings]), and 55.3% in facilities that did not provide influenza vaccination at no cost to employees (range: 50.5% [LTCFs] to 71.7% [hospitals]).

## Editorial Note

The overall HCP influenza vaccination coverage estimate from this opt-in Internet panel survey for the 2012–13 season was 72.0%, an increase compared with the previous two influenza seasons (6,9). Increases in vaccination coverage were observed across all occupation types and in all occupational settings and was highest for two categories of participants in this survey: HCP working in occupational settings with vaccination requirements and physicians (irrespective of the administrative policies of the setting in which they worked). Although increases in influenza vaccination coverage were observed in all occupational settings from the 2011–12 to 2012–13 seasons, coverage during these seasons was lowest among HCP working in LTCF. Among HCP work settings, overall vaccination was highest among HCP working in hospitals.

Ensuring high HCP vaccination coverage each season requires organized efforts by health-care facilities. Appropriate facility policies can help achieve continuing high vaccination coverage during each influenza season. The results of this survey showed that vaccination requirements, vaccination promotion, and access to vaccination at no cost to the HCP for ≥1 days were associated with higher vaccination coverage among HCP. Worksite vaccination, the most common place of vaccination reported by HCP in this survey, has been associated with higher seasonal vaccination coverage among HCP (8); however, this study found that 32% of HCP worked in health-care facilities that either did not offer vaccination on-site, or if offered, did not make vaccination available at no cost. These results indicate that a comprehensive intervention strategy that includes education and promotion to encourage vaccination along with easy access to vaccination at no cost on multiple days might increase HCP vaccination coverage.

Consistent with the prior season, coverage among HCP in LTCF was the lowest among examined occupational settings; coverage remained lower than the 2010–11 estimate, but increased from the 2011–12 estimate. Influenza vaccination of HCP in this setting is extremely important given that influenza vaccine effectiveness is generally lowest in the elderly, making vaccination of close contacts even more critical (2,4). In addition, multiple studies have demonstrated health benefit to patients, including reduced risk for death, with vaccination of HCP in LTCF (1–4). A total of 10.1% of LTCF HCP reported that their facility made vaccine available at no cost for >1 day and 30.5% reported that their facility neither required nor promoted vaccination. In contrast, 58.0% of hospital HCP reported that their facility made vaccine available at no cost for >1 day and 19.4% reported their facility neither promoted nor required vaccination. More efforts are needed to implement evidence-based strategies to increase influenza vaccination coverage among HCP working in LTCF, including promoting vaccination and providing vaccine at low or no cost.

The findings in this report are subject to at least six limitations. First, the findings in this study might differ from those based on the National Health Interview Survey (NHIS), a probability-based survey that might provide better representativeness of the general health-care provider population. Influenza vaccination among HCP from the opt-in Internet panel survey (63.4%) differed from the population-based sample in the NHIS (57.5%) in the 2009–10 season (10). A similar difference (63.5% in the opt-in Internet panel survey versus 55.8% in the NHIS) was observed in the 2010–11 season (9) (Assessment Branch, Immunization Services Division, National Center for Immunization and Respiratory Diseases, CDC, unpublished data, 2012). Additional comparisons with NHIS and other available data sources over multiple seasons are needed to determine whether the more timely opt-in Internet panel survey estimates, despite sampling differences, provide valid assessments of trends. Second, the sample was not randomly selected from the approximately 18 million HCP in the United States. The sample consisted of a nonprobability sample of volunteer HCP members of the Medscape and SurveySpot Internet panels and did not include HCP without Internet access. Despite poststratification weighting, the results based on this nonprobability sample might not be representative of the HCP population in the United States. Third, all results were based on self-report and were not verified by employment or medical records. Self-report of vaccination might be subject to recall bias. Noncoverage and nonresponse bias might remain even after weighting adjustments. Fourth, the definition of vaccination promotion changed in the 2012–13 survey from previous surveys; therefore, the vaccination promotion trend is not comparable across survey years. Fifth, the 2012–13 and 2011–12 opt-in Internet panel survey data might not be directly comparable to the 2010–11 opt-in Internet panel survey data because different methods of recruitment were used in the earlier season. Finally, the definition of HCP, occupation type, and occupational setting used in this opt-in Internet panel survey vary from definitions used in other surveys of vaccination coverage, so that results might not be comparable.

The *Guide to Community Preventive Services* describes evidence-based strategies and recommends interventions with on-site, free, and actively promoted influenza vaccination services to increase vaccination coverage (7). The results of this opt-in Internet survey support expanding the number of health-care facilities offering vaccination on-site, over multiple days, and at no cost as strategies to improve vaccination. Implementing vaccination promotion policies and evidence-based strategies can help sustain and increase HCP influenza vaccination coverage over time.

## Figures and Tables

**FIGURE 1:**
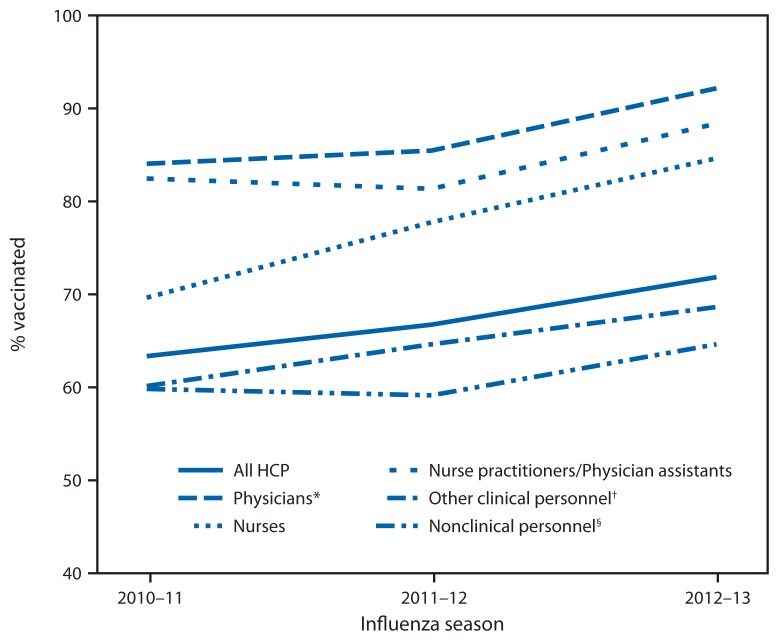
Percentage of health-care personnel (HCP) who received influenza vaccination, by occupation type — Internet panel survey, United States, 2010–11, 2011–12, and 2012–13 influenza seasons * Included dentists in 2010–11 season. ^†^ All seasons include pharmacists, allied health professionals, technicians, technologists, assistants, or aides. Dentists were added starting from the 2011–12 season. ^§^ Administrative support staff or manager, and nonclinical support staff (e.g., food service workers, housekeeping staff, maintenance staff, janitors and laundry workers).

**FIGURE 2:**
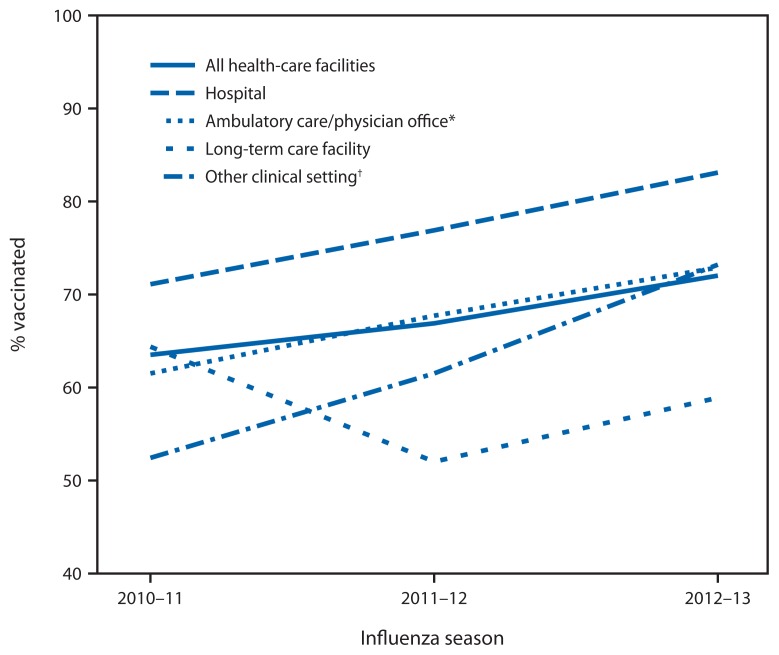
Percentage of health-care personnel (HCP) who received influenza vaccination, by occupational setting — Internet panel survey, United States, 2010–11, 2011–12, and 2012–13 influenza seasons * Ambulatory care (physician’s office, medical clinic, and other ambulatory care setting). ^†^ Including dental offices, pharmacies, nonhospital laboratories, medical-related schools, emergency medical technician sites, and home health-care sites.

**TABLE:** Percentage of health-care personnel (HCP)* who received influenza vaccination, by occupational setting, occupation type, vaccine availability, and requirements status — Internet panel survey, United States, 2010–11, 2011–12, and 2012–13 influenza seasons

	2010–11			2011–12			2012–13		
			
Characteristic	Sample size	Weighted %†	Weighted % vaccinated	Sample size	Weighted %†	Weighted % vaccinated	Sample size	Weighted %†	Weighted % vaccinated
**Overall**	**1,931**	**(100.0)**	**(63.5)**	**2,348**	**(100.0)**	**(66.9)**	**1,944**	**(100.0)**	**(72.0)**
**Occupation type, by occupational setting**									
Physician	430	(4.0)	(84.2)	418	(5.1)	(85.6)	322	(5.6)	(92.3)
Hospital	47	(14.0)	(81.3)	247	(54.7)	(86.7)	209	(59.5)	(93.2)
Ambulatory care/physician office§	359	(79.0)	(86.2)	311	(76.7)	(86.2)	221	(71.1)	(91.6)
Long-term care facility	—¶	—¶	—¶	—¶	—¶	—¶	—¶	—¶	—¶
Other clinical setting**	—¶	—¶	—¶	—¶	—¶	—¶	—¶	—¶	—¶
Nurse practitioner/Physician assistant	72	(3.8)	(82.6)	151	(1.4)	(81.5)	131	(1.6)	(88.5)
Hospital	—¶	—¶	—¶	69	(47.2)	(84.1)	50	(37.9)	(88.0)
Ambulatory care/physician office§	49	(62.0)	(88.4)	103	(69.9)	(83.5)	94	(75.0)	(92.6)
Long-term care facility	—¶	—¶	—¶	—¶	—¶	—¶	—¶	—¶	—¶
Other clinical setting**	—¶	—¶	—¶	—¶	—¶	—¶	—¶	—¶	—¶
Nurse	255	(22.2)	(69.8)	373	(24.4)	(77.3)	202	(22.8)	(84.8)
Hospital	151	(67.5)	(75.4)	252	(59.7)	(78.0)	121	(56.6)	(86.5)
Ambulatory care/physician office§	37	(15.5)	(74.2)	91	(34.6)	(74.4)	48	(28.9)	(79.9)
Long-term care facility	—¶	—¶	—¶	54	(7.3)	(71.4)	32	(8.9)	(85.4)
Other clinical setting**	39	(2.7)	(54.7)	—¶	—¶	—¶	—¶	—¶	—¶
Pharmacist††	—††	—††	—††	—††	—††	—††	92	(0.6)	(89.1)
Hospital	—††	—††	—††	—††	—††	—††	44	(52.4)	(97.7)
Ambulatory care/physician office§	—††	—††	—††	—††	—††	—††	—¶	—¶	—¶
Long-term care facility	—††	—††	—††	—††	—††	—††	—¶	—¶	—¶
Other clinical setting**	—††	—††	—††	—††	—††	—††	61	(65.9)	(88.5)
Other clinical personnel§§	776	(40.0)	(60.3)	980	(40.5)	(64.8)	722	(41.9)	(68.6)
Hospital	243	(38.7)	(71.0)	441	(33.4)	(79.5)	345	(36.2)	(80.6)
Ambulatory care/physician office§	118	(10.4)	(47.1)	157	(20.5)	(73.4)	177	(28.0)	(77.4)
Long-term care facility	120	(19.2)	(63.4)	208	(26.5)	(48.3)	195	(31.2)	(55.3)
Other clinical setting**	295	(31.7)	(53.8)	241	(24.6)	(66.1)	82	(14.7)	(75.0)
Nonclinical personnel ¶¶	398	(30.0)	(60.0)	426	(28.5)	(59.3)	449	(27.2)	(64.8)
Hospital	163	(45.5)	(66.2)	178	(49.2)	(71.7)	177	(41.9)	(79.5)
Ambulatory care/physician office§	95	(17.7)	(52.2)	85	(34.8)	(53.9)	79	(35.6)	(58.6)
Long-term care facility	57	(17.1)	(74.5)	155	(13.5)	(54.4)	165	(11.4)	(60.8)
Other clinical setting**	83	(19.7)	(47.9)	—¶	—¶	—¶	46	(15.3)	(56.7)
**Occupational setting** ***									
Hospital	617	(45.5)	(71.1)	1,187	(45.6)	(76.9)	961	(43.9)	(83.1)
Ambulatory care/physician office§	658	(18.5)	(61.5)	747	(31.6)	(67.5)	636	(33.3)	(72.9)
Long-term care facility	220	(14.7)	(64.4)	455	(16.7)	(52.0)	427	(18.6)	(58.9)
Other clinical setting**	436	(21.3)	(52.4)	277	(12.7)	(61.5)	237	(15.3)	(73.2)
**Influenza vaccination requirement and promotion (2012–13 season definition), by occupational setting**									
Required	230	(20.0)	(98.1)	496	(29.6)	(93.7)	549	(30.0)	(96.5)
Hospital	121	(68.7)	(98.1)	362	(61.4)	(95.2)	388	(62.3)	(95.1)
Ambulatory care/physician office§	76	(15.8)	(96.2)	153	(33.1)	(95.5)	191	(31.9)	(99.8)
Long-term care facility	—¶	—¶	—¶	45	(9.5)	(86.1)	61	(13.0)	(95.8)
Other clinical setting**	—¶	—¶	—¶	—¶	—¶	—¶	38	(7.4)	(100.0)
No requirement, but vaccination promotion†††	320	(17.5)	(64.8)	390	(18.3)	(75.4)	901	(45.9)	(76.9)
Hospital	141	(50.3)	(62.0)	255	(53.3)	(75.4)	456	(44.6)	(78.1)
Ambulatory care/physician office§	88	(14.3)	(60.2)	106	(27.7)	(70.0)	273	(32.8)	(80.1)
Long-term care facility	31	(16.9)	(71.9)	62	(18.9)	(77.7)	183	(16.1)	(67.0)
Other clinical setting**	60	(18.4)	(71.8)	30	(9.6)	(95.0)	134	(19.5)	(85.7)
No requirement or promotion	1,373	(62.4)	(56.7)	1,450	(52.1)	(55.2)	487	(24.1)	(50.4)
Hospital	352	(36.7)	(64.2)	566	(34.6)	(65.0)	115	(19.4)	(67.7)
Ambulatory care/physician office§	490	(20.4)	(56.5)	486	(32.4)	(57.0)	170	(36.2)	(50.4)
Long-term care facility	173	(15.6)	(58.2)	343	(19.0)	(41.4)	179	(30.5)	(45.0)
Other clinical setting**	358	(27.2)	(48.4)	225	(18.9)	(56.5)	65	(17.3)	(50.2)
**Influenza vaccination availability at no cost, by occupational setting**									
>1 day§§§	1,304	(75.6)	(74.8)	1,355	(59.6)	(78.4)	1,079	(54.1)	(86.2)
Hospital	551	(56.4)	(75.8)	899	(58.6)	(80.1)	702	(58.0)	(87.5)
Ambulatory care/physician office§	457	(19.6)	(74.5)	432	(31.1)	(78.8)	332	(33.4)	(88.8)
Long-term care facility	131	(12.9)	(74.5)	143	(8.1)	(62.7)	145	(10.1)	(79.4)
Other clinical setting**	165	(11.1)	(71.2)	99	(9.6)	(88.4)	107	(11.5)	(86.9)
1 day§§§	75	(3.9)	(52.1)	297	(15.0)	(67.7)	304	(14.2)	(75.7)
Hospital	—¶	—¶	—¶	134	(36.9)	(69.6)	126	(40.7)	(76.3)
Ambulatory care/physician office§	—¶	—¶	—¶	105	(34.1)	(64.9)	117	(32.8)	(84.6)
Long-term care facility	—¶	—¶	—¶	53	(19.1)	(59.1)	76	(22.8)	(63.0)
Other clinical setting**	—¶	—¶	—¶	44	(17.0)	(90.5)	34	(13.5)	(87.6)
Not available¶¶¶	543	(20.5)	(41.7)	682	(25.4)	(48.4)	561	(31.6)	(55.3)
Hospital	43	(10.3)	(40.8)	151	(22.1)	(66.8)	133	(21.2)	(71.7)
Ambulatory care/physician office§	180	(13.1)	(30.0)	209	(32.1)	(51.3)	187	(33.5)	(53.3)
Long-term care facility	71	(20.4)	(52.1)	252	(32.5)	(43.2)	206	(31.3)	(50.5)
Other clinical setting**	249	(56.1)	(42.7)	131	(18.1)	(39.8)	96	(22.6)	(62.0)

* Persons who work in a place where clinical care or related services was provided to patients, or whose work involves face-to-face contact with patients, or who were ever in the same room as patients.

† Weights were calculated based on each occupation type, by age, sex, race/ethnicity, occupational setting, and census region, to represent the U.S. population of HCP. Overall occupation type, occupational setting (main heading), requirement, and vaccination availability are presented as weighted estimates of the total sample. Where the groups are stratified by occupational setting, the weighted estimates are presented for each subgroup within the group. The totals for the subgroups will not equal 100% because HCP could specify working in more than one occupational setting.

§ Ambulatory care (physician’s office, medical clinic, and other ambulatory care setting).

¶ Estimate suppressed because sample size was <30.

** Respondents who only reported working in a dentist office or dental clinic; pharmacy; laboratory; public health setting; medical, nursing, or other health-care education setting; emergency medical services setting; or other setting where clinical care or related services were provided to patients.

†† Data on pharmacists only available for 2012–13 season, individual data on pharmacists not collected in prior seasons.

§§ Allied health professional, technician, technologist, assistant, or aide.

¶¶ Administrative support staff or manager and nonclinical support staff (e.g., food service workers, housekeeping staff, maintenance staff, janitors, and laundry workers).

*** Respondents were able to select more than one work setting.

††† Influenza vaccination was promoted among employees through public identification of vaccinated persons, financial incentives or rewards to groups of employees, competition between units or care areas, free or subsidized cost of vaccination, reminder, publicizing of the number or percent of employees receiving vaccination, and special events.

§§§ Question only asked of those reporting influenza vaccinations offered on-site during this influenza season.

¶¶¶ Influenza vaccinations not offered on-site during the influenza season or offered on-site but not available at no cost to employees.

## Footnotes

* Comparable National Health Interview Survey data for this population will not be available until July 2014.

† Additional information available at http://www.surveysampling.com.

§ A survey response rate requires specification of the denominator at each stage of sampling. During recruitment of an online opt-in survey sample, such as the Internet panel described in this report, these numbers are not available; therefore, the response rate cannot be calculated. Instead, the survey completion rate is provided.

¶ Ambulatory/physician office included physician’s offices, medical clinics, and other ambulatory care settings. LTCF included nursing homes, home health agencies, assisted living settings, or other LTCF. Other clinical setting included dental offices or clinics, pharmacies, laboratories, public health settings, medical, nursing, or other health-care education settings, emergency medical services settings, or other settings where clinical care or related services were provided to patients.

** Additional information available at http://www.aapor.org/opt_in_surveys_and_margin_of_error1.htm.

## References

[1] Carman WF, Elder WG, Wallace LA (2000). Effects of influenza vaccination of health-care workers on mortality of elderly people in long-term care: a randomized controlled trial. Lancet.

[2] Hayward AC, Harling R, Wetten S (2006). Effectiveness of an influenza vaccine programme for care home staff to prevent death, morbidity, and health service use among residents: cluster randomised controlled trial. BMJ.

[3] Lemaitre M, Meret T, Rothan-Tondeur M (2009). Effect of influenza vaccination of nursing home staff on mortality of residents: a cluster-randomized trial. J Am Geriatr Soc.

[4] Oshitani H, Saito R, Seki N (2000). Influenza vaccination levels and influenza-like illness in long-term-care facilities for elderly people in Nigata, Japan, during an influenza A (H3N2) epidemic. Infect Control Hosp Epidemiol.

[5] CDC (2011). Immunization of health-care personnel: recommendations of the Advisory Committee on Immunization Practices (ACIP). MMWR.

[6] CDC (2012). Influenza vaccination coverage among health-care personnel—United States, 2011–12 influenza season. MMWR.

[7] Community Preventive Services Task Force (2013). Increasing appropriate vaccination: universally recommended vaccinations. The Guide to Community Preventive Services.

[8] Harris K, Maurer J, Black C, Euler G, Kadiyala S (2011). Workplace efforts to promote influenza vaccination among healthcare personnel and their association with uptake during the 2009 pandemic influenza A (H1N1). Vaccine.

[9] CDC (2011). Influenza vaccination coverage among health-care personnel: 2010–11 influenza season, United States. MMWR.

[10] CDC (2010). Interim results: influenza A (H1N1) 2009 monovalent and seasonal influenza vaccination coverage among health-care personnel—United States, August 2009–January 2010. MMWR.

